# SeqPlots - Interactive software for exploratory data analyses, pattern discovery and visualization in genomics

**DOI:** 10.12688/wellcomeopenres.10004.1

**Published:** 2016-11-15

**Authors:** Przemyslaw Stempor, Julie Ahringer

**Affiliations:** 1 The Gurdon Institute and the Department of Genetics, University of Cambridge, Cambridge, CB2 1QN, UK

**Keywords:** k-means cluster, hierarchical cluster, self-organizing maps, unsupervised machine learning, aggregate gene profile plot

## Abstract

Experiments involving high-throughput sequencing are widely used for analyses of chromatin function and gene expression. Common examples are the use of chromatin immunoprecipitation for the analysis of chromatin modifications or factor binding, enzymatic digestions for chromatin structure assays, and RNA sequencing to assess gene expression changes after biological perturbations. To investigate the pattern and abundance of coverage signals across regions of interest, data are often visualized as profile plots of average signal or stacked rows of signal in the form of heatmaps. We found that available plotting software was either slow and laborious or difficult to use by investigators with little computational training, which inhibited wide data exploration. To address this need, we developed SeqPlots, a user-friendly exploratory data analysis (EDA) and visualization software for genomics. After choosing groups of signal and feature files and defining plotting parameters, users can generate profile plots of average signal or heatmaps clustered using different algorithms in a matter of seconds through the graphical user interface (GUI) controls. SeqPlots accepts all major genomic file formats as input and can also generate and plot user defined motif densities. Profile plots and heatmaps are highly configurable and batch operations can be used to generate a large number of plots at once. SeqPlots is available as a GUI application for Mac or Windows and Linux, or as an R/Bioconductor package. It can also be deployed on a server for remote and collaborative usage. The analysis features and ease of use of SeqPlots encourages wide data exploration, which should aid the discovery of novel genomic associations.

## Introduction

Sequencing based techniques such as ChIP-seq and RNA-seq are widespread experimental tools that generate vast amounts of data for downstream analyses such as uncovering global patterns of genomic activity. After aligning sequence reads to the reference genome, read coverage is calculated. Visualizing coverage tracks using genome browsers is the simplest way to inspect the results. Nevertheless, calculating and plotting signals across groups of selected genomic locations is essential for genome-wide hypothesis testing and quantitative comparisons.

Typically, users plot the abundance of signal (e.g., read coverage) across a set of genomic regions (e.g., transcription start sites) either as a profile plot of average signal or as stacked rows of individual signals visualized as a heatmap. Such plots are usually generated using online or command line tools such as Galaxy/Cistrome, ngs.plot, and deeptools, or using custom scripts combined with plotting software such as Gnuplot ^1– 5^. We found that these methods were either laborious, as each plot needed to be set up individually, or were difficult to use by those with little computational training. These factors inhibited users from generating a large number of plots for data exploration.

To address this, we developed SeqPlots, a highly configurable, graphical user interface (GUI) operated application that rapidly generates publication quality average profile plots or heatmaps that can be clustered using different algorithms to uncover patterns within the data. A key feature of SeqPlots is the ability to select a set of features and signals, then rapidly plot them in any combination, facilitating wide data exploration.

## Methods

SeqPlots can plot signals from any experimental or *in silico* data (e.g. ChIP-seq or RNA-seq read coverage, density of sequence motifs, mappability, nucleosome occupancy) over one or multiple sets of genomic features, (e.g. TSSs, gene bodies, peak calls). Users first add signal tracks and genomic feature files to an integrated SeqPlots database (see Table 1 for accepted file formats). Then any combination of signal and feature files in the database, together with any user entered sequence motifs, can be analyzed. Plots can be anchored at either end of a feature, at both ends, or at centers, and users can define which lengths of upstream and downstream sequence to plot. Additionally, three different methods can be used to cluster heatmaps: k-means, hierarchical clustering, and self organizing maps (unsupervised neural networks); heatmap rows can also be sorted by signal strength.

**Table 1.** File formats accepted by SeqPlots.

**Genomic feature formats**	
**File formats**	**Recognized extensions**
General Feature Format	gff
Browser Extensible Data	bed
General Transfer Format	gtf
**Signal track formats**	
**File formats**	**Recognized extensions**
bigWig Track Format ^a^	bw
Wiggle Track Format ^b^	wig
BedGraph Track Format ^b^	bdg or bedGraph
Binary Sequence Alignment/Map ^c^	bam

^a^preferred track format ^b^converted to bigWig upon upload ^c^coverage is calculated using all aligned reads

## Implementation

SeqPlots utilizes indexing and the multi-layer summarization properties of bigWig files for rapid data acquisition ^6^, and precalculates and stores profiles for all combinations of selected signals and features. Users are presented with a clickable array of signal/feature pairs that can be plotted individually or in any combination in a matter of seconds. Average profile plots or heatmaps are immediately displayed as previews and can be downloaded as PDF files. Profile plots can display standard error and 95% confidence intervals. Spreadsheets with annotated heatmap clusters can be downloaded for downstream analyses such as additional clustering or gene enrichment analyses. Scaling, colors, axes, and titles are also easily configurable. Signal and feature files uploaded to the integrated SeqPlots database are available for use in later plot setups. Users can search and sort uploaded files, and annotate them with comments, user names and reference genome versions.

## Use case

**Figure 1.**
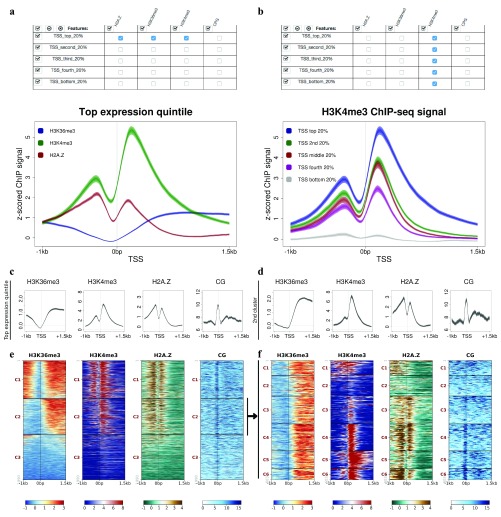
An example of SeqPlots workflow to analyze H2A.Z, H3K36me3, H3K4me3 and CpG density across *C. elegans* protein coding TSSs separated by expression quintiles. ( **a,b**) Top, GUI interface showing clickable grid of signal/feature combinations. Bottom, plots resulting from the clicked selections. ( **c**) Plots of individual signals across genes in top expression quintile anchored at TSSs, plotting 1 kb upstream and 1.5 kb downstream of TSSs. ( **d**) Heatmaps generated using k-means clustering (3 clusters) of TSSs in top expression quintile, using H3K36me3 signal for clustering. ( **e**) Average signal profiles and ( **f**) heatmaps generated from cluster 2 (C2) in ( **d**) made by downloading full cluster data and uploading file with cluster 2 regions. Heatmaps were clustered using H3K4me3, H2A.Z and CpG signals. Data used to generate this figure are available from GEO (H3K4me3: GSE28770 - https://www.be-md.ncbi.nlm.nih.gov/geo/query/acc.cgi?acc=GSE28770; H3K36me3: GSE62833 - https://www.be-md.ncbi.nlm.nih.gov/geo/query/acc.cgi?acc=GSE62833; H2A.Z/HTZ-1: GSE49717 - https://www.be-md.ncbi.nlm.nih.gov/geo/query/acc.cgi?acc=GSE49717). TSS annotations are from 7, 8, or Wormbase/Ensembl 81 if a gene had no TSS annotation in either dataset (available from https://gist.github.com/Przemol/c5114067cc2dd236ed1dbcaf41003472). Genes were divided into expression bins using DCPM values from Gerstein *et al.* ^9^.

Figure 1 illustrates a typical use of SeqPlots. Five feature files in bed format containing genomic coordinates of protein coding genes in different expression bins were selected together with three bigWig signal files (normalized read coverage of H3K4me3, H2A.Z, and H3K36me3). In addition the dinucleotide motif CG was inputted and SeqPlots generated a CG density track for use in the analyses. A plot type anchored at the start position (the TSS) was then selected, and 1 kb upstream and 1.5 kb downstream of the TSS was specified. Following the setup and calculation, SeqPlots presented a clickable grid (top of Figure 1a,b). Selecting the desired combinations and plot type (average profile plot or heatmap) generates a plot. In Figure 1a, three signals (H3K36me3, H3K4me3, and H2A.Z) and one feature (top 20% TSSs) were selected for an average profile plot. For Figure 1b, these were deselected and a new combination was selected (H3K4me3 and all five TSS expression classes). For Figures 1c and d, single combinations of feature and signal were selected. A three-cluster heatmap was then generated using all four signal tracks, clustered using just the H3K36me3 signal. This simple clustering identified regions with bidirectional (C1), unidirectional (C2) or little (C3) H3K36me3. The unidirectional cluster (C2) was extracted from the cluster annotation spreadsheet and uploaded for further re-clustering. A self organizing map with 6 neurons was applied to the three other features – H3K4me3, H2A.Z and CpG, revealing clusters with different patterns of H3K4me3 and H2A.Z marking. For example, cluster C4 shows strong H3K4me3 downstream of the TSS and H2A.Z enrichment both upstream and downstream of the TSS whereas cluster C6 has a similar H3K4me3 pattern, but H2A.Z shows higher enrichment upstream of the TSS. Additionally, clusters C4–C6 have a stronger CpG signal at the TSS than clusters C1–C3. This simple example shows how SeqPlots can be used to find relationships between genomic features and signals. The rapid plotting capability and ease of use of SeqPlots should facilitate wide exploration of high-throughput sequencing data, leading to the discovery of novel biological associations.

## Software availability

SeqPlots is distributed as user-friendly stand-alone applications for Mac and Windows or Linux, and is available as an R programming language package from the Bioconductor repository. SeqPlots can be also deployed as a server application, which is useful for data sharing within laboratories, collaborative usage and remote work. SeqPlots is an open source and open development project: source code wiki, bug tracker and pull requests are available via GitHub.

Software is available from:

• http://przemol.github.io/seqplots (Mac, Windows, Linux, full documentation)
• http://bioconductor.org/packages/seqplots (R/Bioconductor)
• http://przemol.github.io/seqplots/#installation---server-deployment (server deployment)
• https://github.com/Przemol/seqplots (latest source code, open development tools, including wiki, bug tracker, and pull requests)

Archived source code as at the time of publication:

• DOI: 10.5281/zenodo.163638, https://zenodo.org/record/163638 (core R/Bioconductor package) ^10^
• DOI: 10.5281/zenodo.163641, https://zenodo.org/record/163641 (Stand-alone GUI application for Mac, Windows and Linux) ^11^

License: LGPL 2.1: https://www.gnu.org/licenses/old-licenses/lgpl-2.1.html
