# Supplementary material for: SeqPlots - Interactive software for exploratory data analyses, pattern discovery and visualization in genomics
Source: Wellcome Open Res. 2016 Nov 15;1:14. [Version 1] doi: 10.12688/wellcomeopenres.10004.1 (PMC5133382; doi:10.12688/wellcomeopenres.10004.1)
Supplement: Peer review history

# Peer review: Referee response for version 1

**DOI:** 10.21956/wellcomeopenres.10780.r17658

**Published:** 2016-11-30

**Authors:** Jean-Christophe Andrau, Anne Coleno

**Affiliations:** 1 Institute of Molecular Genetics of Montpellier (IGMM) - UMR5535, Montpellier, France

Seqplots is an analysis and visualization software for genomic data sets, including ChIPseq, RNAseq and others. It allows investigating the pattern and abundance of signal coverage across genomic regions. Seqplots provides options for average profiling, performing heatmaps of normalized signal that can be clustered or not and further retrieved to explore the data. Seqplots is accessible as an application for all interfaces (Mac, Windows, Linux) or as R/Bioconductor package. The online documentation presents a dataset of a transcription factor and two epigenetic marks (H3K4me3 and H3K36me3) on the first chromosome of *C. elegans*.

**Installation:**

 To challenge Seqplots, we tested our own data on the complete human genome. The latest version (3.0.12 MacOSX bundle version) of Seqplots was first downloaded on a Mac but yielded genome installation issues. We thus went on with an older version (1.7.16, MacOS bundle version 2.0.2) that contains two drosophila genome versions (dm3 and dm6), *C. elegans* genome ce10 and human hg19 and we proceeded with this one.

**Genomes upload:**

 One can select the desired genome on the proposed list but the action of « install selected » stops half way after indicating « installing packages: BSgenome.Scerevisiae.U ». The blue progression line stops but no error message appears, leaving no option to correct any possibility of upload. There is no indication of genome format file needed to upload, whether it would be FASTA, indexed genome or anything else.

**Data upload:**

 Data (bam, wig, bigwig) can be easily uploaded to Seqplots that automatically converts any format in bigwig to allow better handling. Data upload time is directly dependent on your data size. After upload, the file is directly converted to bigwig.

**New Plot Set:**

 To generate a profile plot or heatmap, one has to chose two files: the file containing the signal intensity (wig, sam …) and the file containing the selected annotations of interest such as genes, protein coding genes, promoters. This second file can be at the bed, gtf or gff format.

 Several files can be processed in a single analysis; the visualization tool uses results to plot individual or combined profiles as selected by the user.

Options to select:

 - Bin size of the signal file has to be known in advance and it is important to adjust this parameter properly, ideally identical to the original data input bin size. If using a bin size below the actual data, it will introduce wholes in the plot. Conversely, using a larger bin size will smooth the data, which can be useful for clarity of the results but can also result in a loss of resolution. This point could be clarified in the documentation. We also note that the link to explanation is essentially inactive.

 - The ‘point view’, ‘end point’ or ‘midpoint’ options are easy to understand as start, middle and end of the features. The ‘anchored features’ option is a bit less obviously accessible, and should be explained more clearly.

Profiles:

 Profiles are easily generated and modified using indicated options, and overall functions well. That the user could directly change the labels below the plots could be a possible improvement.

Heatmaps:

 This option also allows clustering of the data with a nice graphical interface, including nice color options. When the process is complete, this tool is very useful to identify classes of genes/features and to explore the data. However, we note that calculation time for heatmap generation can be limiting and varies quite a lot from one time to the other. Unfortunately, no error message appears to explain if something went wrong on the interface while generating the heatmap. Sometimes the message is blocked in “exporting results” but these are never exported.

**General considerations and conclusion:**

 Seqplots can be used quite easily by non bio-informaticians with minimum training. We believe that this software fulfills many of the analysis options that biologists are looking for when dealing with high-throughput sequencing data sets, including ChIP-seq and RNA-seq. It has therefore a great potential of usage by the community of scientists interested in genomic science. We also found that improvements remain possible and suggest debugging more specifically the following points.

- The genome upload issue has to be fixed.
- Documentation should be developed for the sections ‘genome upload’,
- There is no possibility to run two jobs in parallel. Seqplots could allow the opening of two windows or more.
- Error messages have to be clearer and help the user to make a decision on what should be done.
- Heatmap generation jobs are often aborted during calculations.

We have read this submission. We believe that we have an appropriate level of expertise to confirm that it is of an acceptable scientific standard.

# Peer review: Referee response for version 1

**DOI:** 10.21956/wellcomeopenres.10780.r17652

**Published:** 2016-11-28

**Authors:** Boris Lenhard, Malcolm Perry

**Affiliations:** 1 Institute of Clinical Science, Imperial College London, London, UK

'SeqPlots' is clearly a mature package and it provides a graphical interface to make complex plots from within an web browser. I was able to get the package up and running, and generate plots from my own data very quickly after installation through the GUI, which was intuitively navigable. It is clear that a lot of work has gone into providing an impressive array of options to the user.

 The manuscript itself gives an adequate, if brief, description of the purpose and typical output of the resource. It does not attempt to serve as user manual or tutorial.

 A tutorial is provided as a separate, regularly updated document. The tutorial is very detailed, although a few relative links were broken. My one suggestion would be to provide easy access to the example data through the tutorial - I couldn't find it without digging into the package source. Adding an example line of `run(root='/path/to/ex/data')` in the tutorial would be a simple way to do this.  

 In the future, it may be worth considering developing a scriptable back-end to the package, so that users who are comfortable with R can automate their pipelines.

 Overall, for those who want a graphical interface to make these plots this is a very useful resource. 

 ----

 Note: Malcolm Perry tested the software and wrote most of the above comments.

We have read this submission. We believe that we have an appropriate level of expertise to confirm that it is of an acceptable scientific standard.

# Peer review: Referee response for version 1

**DOI:** 10.21956/wellcomeopenres.10780.r17648

**Published:** 2016-11-25

**Authors:** Simon J van Heeringen

**Affiliations:** 1 Department of Molecular Developmental Biology, Radboud University Medical Center, Nijmegen, Netherlands

SeqPlots is a graphical, interactive tool for exploratory visualization of high-throughput sequencing data. To start with my major point, I regret to say that I can not recommend SeqPlots in its current form. The main issue I have is with installation. This is a common hurdle with bioinformatics software, however, it is an important factor for a software tool. I don't gain any satisfaction from pointing this out, as the author has clearly spent a lot of effort on creating installers for all three major operating systems. However, I was not able to install SeqPlots in any form. I do have to admit here that I am not experienced in R. However, I do use other R modules without problems and SeqPlots is positioned as a tool for those with little computational training.

 I tried the following:

1. Linux AppImage (Ubuntu 14.04)
2. Linux zipped application (Ubuntu 14.04)
3. Linux R installation (two different computers running Gentoo Linux)
4. Windows MSI package (Windows 7)
5. Windows zipped application (Windows 7)

These all failed in different ways:

1. & 2. I get a “Loading, please wait” prompt, with “Seqplots is running: false” on the command line.

         3. After the run() command I get a greyed out, non-responsive webpage.

          4. “Installation has failed” (this was after I had to upgrade .NET to be able to run the installer).

          5. There were so many files (700MB in 10,000+ files) that extracting/copying the application took too much time and broke it off.

 I recommend that the authors simplify installation options and then make sure that these really work. For instance, for Windows, there are three different ways to install the package. It is a lot of work to test and support all these releases. In my case not one out of five options worked. Personally, I can highly recommend bioconda, however, other working options would also be fine.

 Given that I could not use the application I have not been able to asses practical use of SeqPlots with my own data.

 With that out of the way, I have to say that the application (as far as I could judge from the demo and the manuscript) seems to be a useful and interesting tool. Documentation is excellent at first glance, although I haven't practically used it. The software is available at Github and archived via Zenodo, perfect.

 One thing that I wondered is how the authors address reproducibility issues. Are the parameters that were used for an analysis saved somewhere? As it is easy to generate many different plots, it would be good to have a way to keep track of this.

 Some other minor remarks and points:

- The authors mention other tools that are “Slow and laborious” and “difficult to use by investigators with little computational training”. I would much prefer that the authors phrase this more positively. This publication will not be screened for novelty, so no reason to downplay other excellent, widely used tools. What are the key properties that are unique to SeqPlots?
- “Such plots are usually generated using online or command line tools such as Galaxy/Cistrome, ngs.plot, and deeptools, or using custom scripts combined with plotting software such as Gnuplot.” A bit pedantic, but I'm not sure anyone uses gnuplot in the various genomics fields. There are other methods that deserve mention, ggplot and R come to mind, and various other plotting tools that have more or less the same functionality.
- I am not a native English speaker, but I am unsure if “wide data exploration” is an existing concept.
- “Common examples are the use of chromatin immunoprecipitation for the analysis of chromatin modifications or factor binding, enzymatic digestions for chromatin structure assays, and RNA sequencing to assess gene expression changes after biological perturbations.” Here, “factor binding” should probably be “transcription factor binding”?
- Are gzipped files accepted as input (for instance for bed and bigbed)?
- The distinction between the “Methods” and the “Implementation” section is a bit fuzzy to me.
- It would be good to add a LICENSE/COPYING file to the repository with the software license.
- Don't use the rainbow/jet palette as the default color scheme, it has too many shortcomings.

I have read this submission. I believe that I have an appropriate level of expertise to confirm that it is of an acceptable scientific standard, however I have significant reservations, as outlined above.
